# Beyond the Rumen: Current Evidence and Knowledge Gaps in Microbial Diversity and Function Across the Bovine Gastrointestinal Tract

**DOI:** 10.1007/s00284-026-05159-8

**Published:** 2026-09-29

**Authors:** Abiodun Mayowa Akanmu

**Affiliations:** Department of Animal Science, University of Pretoria, Pretoria, South Africa

## Abstract

The bovine gastrointestinal tract (GIT) is a spatially organised microbial ecosystem, but the evidence supporting a whole-tract interpretation is highly uneven. This critical review distinguishes well-established rumen biology from emerging observations in the reticulum, omasum, abomasum, small intestine and hindgut. Comparative studies show strong regional filtering of microbial communities, yet most available datasets are cross-sectional, use digesta rather than mucosa, and infer function from DNA. Consequently, the presence of genes or taxa cannot be equated with active metabolism. Early life provides an important developmental dimension: microbial succession during the milk-to-solid-feed transition accompanies rumen maturation and region-specific immune development, although durable effects on adult productivity remain incompletely demonstrated. Across adult cattle, foregut fermentation supplies most microbially derived energy, whereas downstream compartments support residual fermentation, epithelial interactions and barrier-related processes whose quantitative contributions are less certain. Methanogenesis is therefore treated as one outcome among nutrition, immune function, pathogen resistance and gut integrity. Metagenomics, metatranscriptomics, metaproteomics and metabolomics are complementary rather than interchangeable; coordinated sampling is required to connect functional potential to activity and host phenotype. Priority should be given to longitudinal, multi-compartment, mucosa-and-digesta studies with absolute microbial measurements, metabolite fluxes and transparent causal inference. A tract-wide framework is valuable not because all compartments are equally understood, but because it makes the present evidence imbalance explicit and identifies where microbiome-targeted nutrition can be tested responsibly.

## Introduction

A further reason for caution is that the bovine GIT is not represented by a single, stable microbiome. Community structure varies with diet, age, breed, physiological stage, geography, management and the microhabitat sampled. A meta-analysis of bovine 16 S rRNA gene datasets confirmed strong anatomical differentiation but also exposed substantial study and protocol effects [1]. Accordingly, differences between published studies can arise from genuine biology, technical variation or both. A useful synthesis must identify recurring patterns without presenting every reported taxon as a universal compartment marker [2, 3]. The term microbiome is used here to encompass microorganisms, their genes and their ecological context, while microbiota refers to the organisms themselves. This distinction matters because many cattle studies profile bacterial 16 S rRNA genes and then discuss the entire microbiome. Such studies do not directly measure archaea, fungi, protozoa, viruses, genes, transcripts or metabolites. The present synthesis gives bacterial surveys their appropriate evidential weight but does not treat them as complete representations of microbial function [1, 4, 5].

The bovine GIT converts fibrous feeds into host-available nutrients through microbial and host processes distributed across anatomically distinct regions. The reticulorumen is the dominant fermentation chamber, where bacteria, archaea, anaerobic fungi and protozoa deconstruct plant material and produce volatile fatty acids (VFAs), microbial biomass and gases [6, 7]. This importance has appropriately made the rumen the centre of ruminant microbiology. It has also created a sampling and interpretation bias: knowledge of other compartments is often extrapolated from rumen or faecal data rather than measured directly.

Here, functional integration means the sequential transformation of substrates and microbial products as digesta moves between regions, together with local host–microbe interactions at epithelial surfaces. It does not imply that every region makes an equal quantitative contribution. Comparative surveys show that anatomical region strongly structures microbial composition and gene repertoires, while diet modifies those region-specific patterns [5, 8]. Large genome catalogues also reveal extensive undescribed diversity, but a catalogue of genes remains a map of potential rather than a direct measure of flux [9].

This review therefore has three objectives: to synthesise compartment-specific evidence across the bovine GIT; to integrate developmental, nutritional and health-related functions without allowing methane to dominate the scope; and to grade conclusions as established, emerging or hypothetical. ‘Established’ denotes findings supported by replicated in vivo measurements or convergent studies; ‘emerging’ denotes limited or mainly cross-sectional evidence; and ‘hypothesis’ denotes a mechanistically plausible proposition that still requires direct testing. 

## Discussion

### Evidence Base and Interpretive Framework

Comparative anatomical work has moved the field beyond simple rumen-versus-faeces contrasts. Mao et al. [8] reported compartment-associated bacterial membership and predicted functions, while Lin et al. [5] showed with shotgun metagenomics that regional location explained more variation than diet across ten GIT regions. Holman and Gzyl [1], integrating datasets produced by different laboratories, similarly found clustering by anatomical location. The convergence of these approaches supports a robust conclusion that local selection occurs along the tract. It does not imply that all taxa are restricted to one region or that community boundaries are abrupt. Digesta movement creates dispersal, and adjacent regions share substrates and organisms while differing in retention, pH, secretions and host exposure [1, 5, 8]. The concept of a ‘core’ also needs careful handling. A taxon may be prevalent across animals but vary greatly in abundance or activity, and prevalence thresholds are affected by sequencing depth and taxonomic resolution. Conversely, low-prevalence organisms may encode specialised functions that become important under particular diets. Core functions may therefore be more stable than core taxa, but even functional redundancy has boundaries. Redundant gene annotations do not demonstrate equivalent expression, substrate affinity, growth rate or spatial access [4, 10].

Whole-tract studies have demonstrated regional differentiation. Mao et al. [8] characterised bacterial membership and predicted functions across dairy-cattle GIT sites, and Lin et al. [5] used shotgun metagenomics across ten regions to show that region was the principal deterministic driver, with diet acting as a modifier. These studies support spatial organisation. They do not, by themselves, quantify the net contribution of each region to animal energy supply, methane release, immune status or production. Such outcomes require measurements of substrate flow, absorption, gas exchange, host physiology and microbial activity. The evidence is additionally asymmetric within regions. Rumen samples are numerous and frequently longitudinal; omasal and abomasal observations are sparse; small-intestinal samples are difficult to collect from healthy animals; and rectal or faecal samples are commonly treated as proxies for the entire distal gut. Faeces are useful for repeated non-invasive sampling but cannot recover spatially localised mucosal communities or conditions in the caecum and proximal colon. Thus, ‘beyond the rumen’ is best treated as a research framework, not as evidence that downstream functions are already resolved [1, 5, 8].

### Methodological Constraints

The first methodological decision is the sampling unit. Rumen liquid obtained through an oral tube, cannula liquid, squeezed digesta, whole contents, particles and epimural biopsies capture different communities. The same principle applies downstream. Ileal digesta cannot represent ileal mucosa, and faeces cannot represent the caecal wall. Studies should report the sampled phase, location, time relative to feeding, preservation interval and whether samples were rinsed. Without these details, biological comparisons may be overwhelmed by differences in what was physically collected [3, 11].

Contamination is especially consequential in low-biomass compartments. Reagent-derived DNA and carry-over from high-biomass digesta can produce apparently plausible taxa in the abomasum or small intestine. Extraction blanks, field blanks, mock communities and biomass estimates are therefore not optional quality-control extras. They are part of the evidence needed to decide whether a detected organism is a resident, a transient passenger or a technical contaminant. Statistical contaminant removal should be reported transparently because aggressive filtering can also remove genuine low-abundance organisms [12, 13]. Relative-abundance data create a second interpretive problem. If one population expands, the calculated relative abundance of another can fall even when its absolute cell number is unchanged. This is important after diet shifts, during diarrhoea and across compartments with markedly different biomass. Quantitative PCR, flow cytometry, spike-in standards or total DNA measurements can provide an absolute denominator. At minimum, authors should avoid describing relative changes as microbial growth or suppression unless absolute evidence is available [14, 15]. Sequencing and annotation introduce additional biases. Marker-gene primers differ in coverage, variable regions produce different taxonomic resolution and copy-number variation distorts abundance. Shotgun sequencing reduces some of these limitations but remains sensitive to host DNA, assembly thresholds and reference-database representation. Metagenome-assembled genomes have expanded cattle microbial resources [9, 16], yet incomplete assemblies can split pathways or miss plasmids and strain variation. Functional labels based on distant homology should be interpreted as putative until experimentally validated [3, 11].

Multi-omics studies are not automatically integrative. Samples collected at different times can link genes to metabolites that arose under different conditions, and correlations across a small number of animals are vulnerable to overfitting. Feed intake, particle passage, rumen volume, milk yield and host genotype can drive both microbial and production variables. Models therefore need appropriate repeated-measures structure, external validation and correction for high-dimensional testing. A network edge is a hypothesis about association, not evidence of metabolite exchange [12, 13]. Sampling methods can also alter the biological question. Digesta reflects luminal and particle-associated organisms, whereas biopsies and mucosal scrapings enrich organisms interacting with epithelium. Cross-compartment comparisons are confounded when sample types, DNA extraction protocols, sequencing depths or reference databases differ. Slaughter studies enable broad anatomical coverage but are cross-sectional and may introduce post-mortem changes. Cannulation supports repeated sampling but is invasive and usually limited to one or two sites. Carry-over of digesta also creates a distinction between organisms detected downstream and organisms that are metabolically active there [3, 11, 13].

Shotgun metagenomics resolves genes and genomes more effectively than marker-gene surveys, but DNA persists after activity ceases and gives limited information about transcription, protein abundance or reaction rates [3]. Metatranscriptomics measures expressed RNA but is sensitive to rapid turnover and handling; metaproteomics is closer to catalytic machinery but has peptide-to-taxon ambiguity; metabolomics measures products shared by microbial and host pathways but often lacks source attribution. Predictive ecological models are further constrained by compositional data, unmeasured feed intake and passage, and correlations that do not establish causality [17]. The strongest designs integrate these layers with absolute microbial load, physicochemical measurements and host outcomes.

### Spatial Organisation in Adult Cattle

#### Reticulum and Rumen

The rumen is spatially heterogeneous. Feed particles support adherent cellulolytic and fibrolytic organisms, the liquid phase contains planktonic and recently detached cells, and the epithelium supports an epimural community exposed to host tissue and steep chemical gradients. Protozoa can harbour associated methanogens, while anaerobic fungi penetrate lignified plant tissues and create physical entry points for other degraders. These relationships mean that a rumen sample homogenised from one phase may obscure the ecological organisation that governs substrate access and interspecies hydrogen transfer [4, 18]. Bacteroidota and Bacillota commonly dominate bacterial sequence datasets, but phylum-level summaries conceal marked functional diversity. Members of *Prevotella* differ in polysaccharide, peptide and protein utilisation, and fibre degradation is distributed among several bacterial and fungal lineages. Methanogenic archaea are less abundant than bacteria yet control an important terminal electron-disposal route. Protozoal predation alters bacterial turnover and nitrogen recycling, while bacteriophages may influence population dynamics and gene exchange. Most cattle surveys profile only bacteria and archaea, so the contribution of eukaryotic and viral components remains comparatively under-resolved [7, 19, 20].

Diet changes both resource supply and physicochemical conditions. Rapidly fermentable starch can increase acid production, favour amylolytic and lactate-associated organisms and reduce niches for pH-sensitive fibrolytic populations. High-forage diets provide structurally complex substrates and longer chewing and buffering patterns. Responses are not determined by forage-to-concentrate ratio alone; processing, starch degradability, lipid type, feeding frequency and selective intake also matter. The same formulated diet can therefore generate different rumen conditions among cows, contributing to individual microbial variation [4, 21].

The reticulum receives far less microbiome-specific attention despite its continuous exchange with the rumen. It participates in mixing, sorting and regurgitation, and reticular pH is increasingly used as a practical proxy for ruminal acid-base conditions. Nevertheless, reticular sensors and samples do not necessarily reproduce the range of microenvironments within the rumen mat, ventral liquid and epithelium. Studies should identify the measurement site rather than using rumen and reticulum interchangeably [7, 19, 20]. The reticulum and rumen exchange digesta continuously and function as a coupled fermentation system. The rumen contains diverse bacterial, archaeal, fungal and protozoal guilds that hydrolyse cellulose, hemicellulose, starch, protein and lipids. Global surveys identify a recurrent core community, but relative abundance varies with diet and host [4]. Genome reconstruction has greatly expanded the recognised metabolic repertoire and carbohydrate-active enzyme (CAZyme) diversity [16]. Stable outputs can coexist with taxonomic variation because different organisms encode overlapping pathways, although redundancy should not be assumed without gene-expression or flux evidence.

#### Omasum and Abomasum

The omasum changes the physical and chemical context of digesta through selective retention, particle reduction, water and electrolyte absorption and continued VFA absorption. Because it receives digesta intermittently from the reticulum, its microbiota is expected to include both transported rumen organisms and populations capable of persisting under omasal conditions. Detection of fibre-degradation genes does not demonstrate that substantial fibre digestion occurs locally. Quantitative substrate disappearance across the organ and activity measurements would be needed to separate continued fermentation from carriage of rumen-derived biomass and DNA [1, 5, 8]. Omasal sampling is itself a source of uncertainty. Samples collected through the reticulo-omasal orifice may be used to estimate nutrient flow but do not necessarily represent organisms attached to omasal laminae or epithelium. Slaughter sampling improves anatomical access but loses repeated temporal information. Few studies pair omasal digesta with mucosal tissue and local metabolites, leaving the role of epithelial-associated organisms largely undefined. This is one of the clearest evidence gaps behind broad claims of whole-GIT functional integration [1, 5, 8].

The abomasum acts as the true gastric compartment. Hydrochloric acid and pepsin digest feed and microbial protein, converting rumen microbial biomass into nutrients subsequently absorbed in the small intestine. From the host perspective, microbial death is therefore a productive step. From a microbiome perspective, however, it complicates interpretation because abundant microbial DNA may reflect material being digested rather than an active abomasal community. RNA-based activity measures, microscopy and mucosal localisation are particularly important at this site [1, 5, 8]. The omasum is a selective-flow and absorptive compartment rather than a second rumen. Reduced substrate availability, water removal and particle retention create an ecological transition. Detected microbes may include rumen-derived passengers alongside locally persistent populations; current studies do not adequately separate these possibilities. The abomasum imposes low pH and host enzymatic digestion. Its microbiota is lower in biomass and is shaped by acid tolerance, passage and mucosal association. Evidence for substantial local fermentation in either compartment is limited; their importance may lie more in microbial selection, lysis and metabolite exposure than in bulk VFA production [5, 8].

#### Small Intestine

The small intestine is the principal site for enzymatic digestion and absorption of amino acids, glucose, fatty acids, minerals and vitamins. Microbial density is lower than in the rumen and colon, but proximity to the epithelium gives local organisms potential regulatory importance. The duodenum is exposed to acidic digesta, bile and pancreatic secretions; the jejunum supports intensive absorption; and the ileum provides a transition towards the densely populated large intestine. Treating these regions as one ‘small-intestinal microbiome’ can therefore conceal substantial ecological and immunological differences [5, 22, 23]. Mucosal and luminal communities respond to different selection pressures. Luminal organisms are strongly influenced by incoming digesta and rapid transit, whereas mucosal populations encounter mucus, antimicrobial peptides, immunoglobulins and locally available oxygen. In calves, paired analyses show that mucosal and digesta communities are distinct ([22], 2019). This distinction has practical consequences because luminal sequencing may be suitable for nutrient-transformation questions, while barrier and immune questions require epithelial or mucosal measurements [24, 25].

Bile acids are both digestive molecules and ecological selectors. Conjugated bile entering the duodenum can inhibit susceptible organisms, while microbial deconjugation and transformation alter the bile-acid pool. In cattle, the responsible species, their regional activity and the consequences for host signalling are less resolved than in humans and laboratory animals. Cattle-specific conclusions should therefore be based on measured bile metabolites and genes rather than transferred wholesale from monogastric models [5, 22, 23]. The ileum is increasingly studied during nutritional or inflammatory challenges. Jiang et al. [25] found that a *Saccharomyces cerevisiae* fermentation product altered ileal bacterial profiles and associations with epithelial structure and immune transcripts during feed restriction. The study illustrates how downstream microbiology can be connected to host tissue, but it also illustrates present limitations: slaughter endpoint sampling, small groups and correlations between taxa and transcripts. Replication across challenge models is required before particular organisms can be labelled consistently beneficial [24, 25].

Small-intestinal microbial metabolism may contribute vitamins and other metabolites, but nutritional availability depends on where production occurs relative to absorption and on competition between host and microbes. Metagenomic reconstruction has identified microbial capacity for B-vitamin and menaquinone biosynthesis across ruminant GIT datasets [24]. These findings expand functional hypotheses but do not establish net vitamin supply. Expression, metabolite concentrations, absorption and dietary sufficiency must be measured together [5, 22, 23]. The duodenum, jejunum and ileum combine rapid flow, bile acids, pancreatic enzymes, oxygen gradients near the mucosa and high host nutrient absorption. Regional and age-dependent mucosal immune transcription has been demonstrated in calves [26], and neonatal studies reveal distinct mucosal and digesta communities ([22], 2019). However, most functional claims remain emerging because biomass is low, sites are difficult to access and DNA from foregut organisms may pass through. Plausible local roles include bile transformation, competition with pathogens, production of signalling metabolites and modulation of epithelial maturation, but each requires direct activity and host-response measurements.

#### Caecum, Colon and Rectum

The caecum and proximal colon provide longer retention and a strongly anaerobic environment for substrates escaping foregut and small-intestinal digestion. These include resistant starch, structural carbohydrate, microbial residues, sloughed epithelial material and mucins. Fermentation can recover energy as VFAs, but the nutritional value depends on absorption before excretion. Because microbial biomass produced in the hindgut is largely lost in faeces, fermentation of nitrogenous substrates does not provide the same amino-acid benefit as rumen microbial protein digested in the small intestine [1, 27, 28].

Large-intestinal genome catalogues demonstrate that faecal communities contain extensive uncharacterised bacterial diversity and metabolic capacity. Teseo et al. [27] reconstructed the phylogeny and metabolism of the cattle large-intestinal bacterial community from 436 faecal metagenomes across six countries. This resource supports comparative and functional discovery, but its authors appropriately describe faeces as a proxy. It cannot determine where within the large intestine a genome is most abundant or active, and it gives limited information on mucosal association [5, 8]. High fermentable-carbohydrate flow to the hindgut may lower pH and alter epithelial exposure to organic acids and microbial products. This can occur when grain feeding overwhelms ruminal or small-intestinal starch digestion. Rumen and hindgut responses should not be assumed to move in parallel because the amount of substrate escaping the rumen depends on processing, passage and ruminal adaptation. Paired compartment studies with nutrient-flow measurements are needed to determine whether faecal changes reflect rumen dysbiosis, altered substrate escape or independent distal responses [5, 8].

Faecal samples remain valuable because they can be collected repeatedly, at scale and without surgery. They are suitable for monitoring temporal stability, detecting pathogens and antimicrobial-resistance genes, and developing biomarkers of distal output. Their limitations should be built into study design rather than used to dismiss them. A biomarker can be useful without representing the whole tract, provided it is validated against the outcome it is intended to predict. Problems arise when faecal associations are presented as mechanisms occurring in the rumen, caecum or mucosa without direct evidence [1, 27, 28]. The large intestine receives fibre, starch and endogenous substrates that escape upstream digestion. Anaerobic fermentation produces VFAs, especially acetate, propionate and butyrate, which can be absorbed; nevertheless, foregut VFAs dominate total ruminant energy supply [29]. The hindgut also provides a dense setting for microbial interactions relevant to epithelial integrity and pathogen exclusion. Regional surveys support a community distinct from the foregut [5, 8], but faecal profiles should not be treated as interchangeable with caecal or colonic communities.

Methanogens and methanogenesis genes can occur downstream, but their presence does not quantify net hindgut methane. Gas may be absorbed, used locally, expelled rectally or confounded with eructated rumen methane in whole-animal chambers. Direct compartment-specific flux data are scarce. Hindgut methanogenesis should therefore be described as biologically plausible and incompletely quantified, not as a known major fraction of enteric emissions [1, 27].

The compartment-specific ecological synthesis and its principal evidence gaps are summarised in Table 1.

**Table 1:** Anatomical compartments, dominant ecological features and evidence gaps

Region	Environment	Prominent microbiota	Main substrates	Established/likely functions	Key gaps
Rumen	Anaerobic; buffered, commonly near pH 5.5–7; long retention	Bacteria, archaea, protozoa, anaerobic fungi	Fibre, starch, protein, lipids	Primary fermentation; VFAs; microbial protein; H₂ and CH₄	Strongest evidence; liquid, particle and epithelium niches remain unevenly sampled
Reticulum	Closely coupled to rumen; mixing and particle sorting	Broadly rumen-like assemblages	Shared reticulorumen digesta	Mixing, regurgitation and continued fermentation	Often pooled with rumen; local activity rarely separated
Omasum	Water removal; selective particle flow; declining substrate	Transitional, lower-biomass community	Residual fermentation products and particles	Absorption and microbial selection/turnover	Few direct activity studies; passengers versus residents unresolved
Abomasum	Acidic; pepsin; rapid chemical transition	Acid-tolerant, transient and mucosa-associated taxa	Microbial biomass and host secretions	Microbial lysis; protein digestion; epithelial interaction	Low biomass and contamination risks; local fermentation not quantified
Small intestine	Bile, enzymes, faster flow; mucosal oxygen gradient	Lower-biomass, region-specific mucosal and luminal taxa	Dietary and microbial nutrients; bile acids	Host digestion and absorption; signalling, barrier and immune interactions	Access difficult; calf evidence exceeds adult evidence; activity undermeasured
Caecum	Anaerobic entry to hindgut; residual substrates	Dense fermentative bacterial and archaeal community	Escaped fibre/starch; endogenous material	Secondary fermentation; VFAs; nitrogen transformations	Few longitudinal samples; quantitative flux uncertain
Colon	Anaerobic; progressive water removal	Dense, spatially structured community	Residual and host-derived substrates	Secondary fermentation; barrier and colonisation resistance	Mucosa and lumen often conflated; gas fate unresolved
Rectum/faeces	Terminal output; oxygen exposure after voiding	Terminal luminal community	Residual biomass and metabolites	Useful repeated proxy for distal output	Not a proxy for the entire GIT; handling strongly affects profiles

pH values vary with diet, time after feeding and sampling location

Table synthesises from Mao et al. [8, 22, 23] and Lin et al. [5].

### Early-life Assembly and Developmental Transitions

#### Neonatal Colonisation

The neonatal GIT undergoes rapid ecological succession rather than a simple linear increase in diversity. Facultative anaerobes can initially consume oxygen and create conditions favourable to strict anaerobes, while milk supplies lactose, fat, protein and host-derived bioactive compounds. Maternal skin, vaginal and faecal contact, colostrum, housing, feeding equipment and other calves provide inocula. The relative importance of these sources varies among management systems, making farm a major unit of biological and statistical variation [30–32].

Microorganisms and fermentation-associated genes are detected in the rumen before calves consume substantial solid feed. Jami et al. [31] documented age-associated rumen succession from one-day-old calves to adults, and Rey et al. [32] showed sequential establishment before and during the introduction of solid feed. Early detection does not mean the neonatal rumen is functionally equivalent to the adult organ. Substrate supply, rumen volume, epithelial development, motility and absorption are still changing, so the same taxon may occupy a different ecological role at different ages [22, 33]. Colostrum shapes the host environment as well as microbial exposure. Immunoglobulins, leukocytes, nutrients and antimicrobial factors influence pathogen defence and mucosal maturation. Failure of passive transfer can therefore confound associations between microbiota and disease. Calf microbiome studies should report colostrum timing, quantity and quality, antimicrobial treatments and feeding system. Without these variables, differences attributed to microbes may instead reflect immunity or management [22, 33].

Colonisation begins around birth and proceeds rapidly under the influence of maternal contact, colostrum, milk, environment, medication and management. Perinatal sampling indicates that intestinal communities are dynamic and body-site specific [33]. In neonatal calves, mucosal and digesta communities differ, warning against treating faeces as a complete representation of developing intestinal ecology [22]. Early studies are vulnerable to low-biomass contamination and strong farm effects, so reported ‘core’ neonatal taxa should be interpreted cautiously.

#### Milk-to-solid-feed Transition and Rumen Maturation

Starter intake changes the ecological resources available to the developing rumen. Fermentation of carbohydrate produces VFAs, among which butyrate and propionate are associated with epithelial growth and metabolic development. Physical fibre contributes to rumen motility, muscular development and chewing behaviour, although overly coarse or poorly digestible forage can displace starter intake. The microbial transition is consequently linked to the quantity and quality of both concentrate and forage, not simply to chronological age [34, 35].

Weaning combines nutritional, behavioural and social changes. Abrupt removal of milk may reduce energy intake and temporarily disrupt growth, while gradual weaning can allow starter intake to increase before milk removal. Meale et al. [34] found that weaning altered ruminal and faecal microbiomes, whereas the tested weaning strategies produced less marked differences. This should not be interpreted as proof that weaning method never matters. Outcomes depend on age, starter intake, milk allowance, health and the interval after weaning at which the community is measured [36, 37]. Manipulation during this window is attractive because communities may be less resistant to change. However, direct inoculation has not consistently reprogrammed the calf rumen. Bu et al. [36] reported limited effects of repeated adult-rumen inoculation on calf prokaryotic composition and predicted function. More recently, longitudinal twin work showed that early rumen inoculation can be followed through faecal community development but does not remove strong age-related succession [37]. These results suggest that substrate and host development may constrain introduced organisms more strongly than inoculum availability [34, 35].

Persistence is the critical test of early-life programming. An intervention can alter the microbiota during supplementation yet disappear after diets converge. Demonstrating programming requires untreated follow-up, repeated functional measurements and adult outcomes such as intake efficiency, milk yield, methane or disease incidence. Studies also need to distinguish a persistent microbial change from a persistent host change that subsequently shapes the microbiota. The direction of causality may be bidirectional [36, 37]. Solid feed introduces fermentable plant substrates, increases VFA production and supports papillary and functional rumen development. Weaning is associated with coordinated shifts in ruminal and faecal microbiomes, whereas the precise weaning strategy may have smaller or context-dependent effects [34]. The calf GIT also transforms anatomically, physiologically and immunologically across the pre- to post-weaning period [35]. These findings establish developmental succession, but they do not show that a single early taxonomic state is universally optimal.

#### Immunity, Health and Long-term Programming

The intestinal immune system must develop tolerance to commensals while remaining responsive to pathogens. This involves epithelial tight junctions, mucus, antimicrobial peptides, innate receptors and adaptive immune components. Liang et al. [26] demonstrated that mucosal immune development differs by small-intestinal region and age. Consequently, a microbial exposure may have different effects in the duodenum and ileum or at one and six weeks of age. Faecal profiling alone cannot capture this regional host response [26, 38]. Calf diarrhoea is a syndrome with infectious, nutritional and management causes. Microbial community disruption may precede disease, result from altered intake and fluid loss, or follow antimicrobial treatment. Oikonomou et al. [39] linked faecal microbial features, health and growth in pre-weaned calves, while later cohort studies have reinforced the possibility of microbiota-based risk markers. Nevertheless, diagnostic models require prospective sampling before clinical signs, pathogen testing and external validation across farms. A diversity decline during diarrhoea is not, by itself, evidence that restoring diversity will cure the disease [39–41].

Starter feeding can influence both microbiota and host barrier-related gene expression during weaning [38]. This type of paired host–microbe measurement is more informative than taxonomy alone, but mechanistic interpretation still requires caution. Diet directly affects epithelial metabolism and microbial composition, so parallel changes do not prove mediation. Mediation analysis, metabolite measurements and controlled perturbations can help separate direct nutritional effects from microbiome-dependent pathways [26, 38]. The developing microbiome coincides with region-specific maturation of mucosal immune pathways [26]. Reviews and primary studies link early-life ecology to diarrhoeal risk, barrier development and immune education [42–45]. Health-associated faecal differences in pre-weaning heifers further support biomarker potential, but predictive performance across farms remains to be demonstrated [41]. Evidence that early interventions permanently program adult feed efficiency, methane emission or milk production is promising but not yet causal and generalisable. Longitudinal trials must follow the same animals from birth through production and distinguish persistent effects from diet, housing and age.

### Functional Integration Across Compartments

#### Carbohydrate Fermentation and Host Energy

Plant cell walls contain cellulose, hemicellulose, pectin and lignin arranged in structures that no single microbial species can completely degrade. Primary degraders attach to particles and release oligosaccharides, while other organisms consume sugars and fermentation intermediates. This division of labour supports acetate, propionate and butyrate production but also creates competition. Rapid uptake of released sugars can reduce the return to the primary degrader, whereas removal of inhibitory products can increase overall degradation. Community function therefore emerges from both cooperation and competition [6, 7, 46]. VFA profiles reflect substrate, pathway use, microbial growth and absorption. Propionate is the principal gluconeogenic precursor, acetate supports lipogenesis and butyrate is extensively metabolised by rumen epithelium. Yet molar proportions do not quantify total energy recovered. A diet can alter rumen volume, dilution and absorption so that concentration and production move differently. Studies connecting microbiomes to host energy should use production or flux estimates where possible and report intake and passage [21, 29].

Carbohydrate escaping the rumen is not necessarily wasted. Starch digested enzymatically in the small intestine can provide glucose, while fibre and resistant starch entering the hindgut can be fermented. The optimal site of digestion depends on digestive capacity, animal state and the consequences of substrate overflow. Excessive hindgut fermentation may lower pH and increase epithelial exposure to microbial products. Thus, strategies to increase rumen bypass should not be judged only by rumen disappearance; whole-tract digestibility and gut-health outcomes are required [6, 7, 46]. Carbohydrate degradation forms the nutritional foundation of the microbial contribution to cattle. Rumen CAZymes initiate the breakdown of recalcitrant plant polymers, while cross-feeding distributes sugars, lactate, succinate, hydrogen and other intermediates among guilds. Absorbed acetate, propionate and butyrate support lipogenesis, gluconeogenesis and epithelial metabolism. Residual carbohydrate reaching the hindgut undergoes secondary fermentation, but faecal metabolite concentrations alone cannot estimate production because absorption and transit intervene [6, 29, 46].

#### Protein, Nitrogen and Microbial Biomass

Rumen nitrogen metabolism links dietary protein, non-protein nitrogen, energy availability and microbial growth. Proteolytic organisms release peptides and amino acids, deamination generates ammonia, and microbes assimilate nitrogen when fermentable energy and suitable carbon skeletons are available. Asynchrony can increase ammonia absorption and urea synthesis, raising urinary nitrogen losses. Microbiome associations with nitrogen efficiency should therefore be interpreted alongside rumen ammonia, microbial protein flow, plasma or milk urea and excreta partitioning [6, 7]. Urea recycling provides an important host–microbe connection. Urea can enter the rumen through saliva or across the epithelium and be hydrolysed by microbial ureases. The resulting ammonia can support microbial protein synthesis when fermentable substrate is available. Similar transformations occur downstream, but nitrogen captured in hindgut microbial biomass is poorly recovered as amino acids by the host. The site of microbial synthesis is therefore as important as the biochemical pathway itself [6, 7].

Metagenomic detection of proteases, peptidases, ureases or ammonia-assimilation genes indicates capacity, not net nitrogen flow. Expression and enzyme activity depend on diet and competition, while several organisms can encode the same function. Isotope-labelled nitrogen, omasal flow markers and microbial-purine estimates can connect sequence information to actual transfer. Without these measurements, correlations between taxa and nitrogen efficiency should be framed as candidate mechanisms [6, 7]. In the rumen, proteolysis, peptide use, deamination and microbial protein synthesis determine the flow of amino acids to the small intestine. The host subsequently digests microbial and undegraded feed protein. Downstream microbes transform residual nitrogen and host secretions, yet some microbial protein produced in the large intestine is lost in faeces rather than digested and absorbed. A whole-tract account must therefore distinguish biochemical transformation from nutritional recovery by the host [6, 7].

#### Lipid, Bile and Epithelial Interactions

Dietary lipids are hydrolysed in the rumen, and unsaturated fatty acids undergo extensive biohydrogenation through pathways distributed among interacting organisms. Intermediate fatty acids can influence milk-fat synthesis and microbial membranes. The community response depends on fatty-acid chain length, degree of unsaturation, physical protection and dose. Lipid supplements that reduce methane may also inhibit fibre digestion or alter milk fat, so environmental and production outcomes must be considered together [7, 24, 25]. After the abomasum, pancreatic lipase and bile facilitate digestion and absorption. Microbial bile-salt hydrolases and other transformations could change local toxicity and signalling, but cattle-specific evidence remains fragmentary. Gene presence in metagenomes cannot establish whether sufficient substrate is encountered or whether the products reach host receptors. Targeted bile-acid profiling across intestinal regions, paired with expression and epithelial responses, is a priority for moving this topic beyond extrapolation [7, 24, 25].

Microbial metabolites can influence barrier function through epithelial energy supply, mucus production and immune signalling. Butyrate is frequently discussed as beneficial, yet its effect depends on concentration, site and epithelial state. Likewise, lipopolysaccharide measurements can reflect microbial biomass, lysis, permeability and assay limitations. Studies should avoid reducing barrier biology to one metabolite or one taxonomic group. Multiple host measurements, including tight-junction proteins, permeability markers, histology and inflammatory responses, are needed [7, 24, 25]. Rumen lipolysis and biohydrogenation alter dietary fatty acids before absorption. In the small intestine, host lipases and bile dominate lipid digestion, while microbial bile transformations may influence local ecology and host signalling. These functions are established in general gastrointestinal biology but remain comparatively under-characterised in cattle at species and reaction-rate levels. Mucosal studies, targeted bile-acid metabolomics and isotope tracing are needed before assigning production effects to particular taxa [7, 24].

#### Hydrogen Flow and Methanogenesis

Methanogenesis is thermodynamically connected to fermentation because removal of hydrogen supports reoxidation of microbial cofactors. Hydrogen is not a freely mixed pool: transfer can occur between organisms in close physical association, including protozoa and associated methanogens. Dissolved hydrogen concentration, production rate and access to alternative acceptors influence which pathways are favourable. Archaeal abundance is therefore an incomplete predictor of methane output, and expression of methanogenesis genes or direct gas measurement is more informative [47–49]. Methanogen communities are comparatively conserved at broad taxonomic levels, but strain-level differences and substrate use matter. Hydrogenotrophic pathways dominate in the rumen, while methylotrophic routes use methyl compounds with hydrogen. Dietary interventions can reduce methane by directly inhibiting methanogens, lowering hydrogen production, redirecting reducing equivalents or changing intake and passage. These mechanisms have different implications for fermentation efficiency and adaptation [50, 51].

Alternative hydrogen sinks are sometimes presented as simple replacements for methane, but their capacity and nutritional consequences vary. Propionate formation captures reducing equivalents and supplies a glucogenic VFA. Reductive acetogenesis competes poorly with methanogenesis under typical rumen conditions, although interest continues in conditions that might improve its competitiveness. Nitrate can act as an electron acceptor but introduces nitrite-toxicity risk and requires careful adaptation. A credible mitigation strategy must show where hydrogen is redirected rather than merely documenting lower methane [47–49]. Most enteric methane is eructated from the reticulorumen. Rectal gas and absorbed hindgut methane are difficult to partition in respiration-chamber or tracer measurements. Detection of methanogens in caecal, colonic or faecal samples confirms potential but not emission magnitude. Until direct partitioning methods are validated, numerical claims about the hindgut share should be avoided. This limitation is central to the review’s distinction between microbial potential and system-level outcome [50, 51].

Hydrogen links fermentation pathways. Methanogenic archaea consume H₂ and CO₂, helping maintain favourable redox conditions, while alternative sinks include propionate formation, reductive acetogenesis, nitrate or sulphate reduction and biohydrogenation [48, 49]. Community composition alone is insufficient because hydrogen transfer can occur at short spatial scales and respond rapidly to diet. Methane is predominantly generated in the reticulorumen; the quantitative hindgut contribution remains unresolved. Consequently, interventions should be evaluated for whole-animal emissions, fermentation shifts, intake, productivity, persistence and safety rather than inferred from archaeal abundance alone.

The major pathways, products, host outcomes and limits of inference are summarised in Table 2.

**Table 2:** Processes, pathways and host outcomes across the tract

Process	Representative pathway	Products	Principal site(s)	Host outcome	Evidence boundary
Plant-polysaccharide degradation	Glycoside hydrolases and polysaccharide lyases	Sugars and oligosaccharides	Rumen ≫ hindgut	Substrates for VFA production	Gene inventories strong; in vivo rates and attribution less complete
VFA formation	Acetate, propionate and butyrate pathways	VFAs, CO₂, H₂	Rumen; hindgut secondary	Major energy supply; epithelial effects	Concentrations do not equal production or absorption flux
Protein and N turnover	Proteolysis, deamination, ammonia assimilation	Microbial protein, ammonia	Rumen; intestine downstream	Amino-acid supply; N efficiency	Large-intestinal microbial protein has limited host recovery
Lipid transformation	Lipolysis and biohydrogenation	Modified fatty acids	Rumen; absorption in small intestine	Milk/meat fatty-acid profile	Taxon-to-reaction assignments remain incomplete
Hydrogen disposal	Methanogenesis and alternative electron sinks	CH₄ or reduced products	Predominantly reticulorumen	Redox balance; energy loss; emissions	Hindgut CH₄ flux not adequately quantified
Barrier and immune interaction	Microbial metabolites and pattern recognition	Signals and immune responses	Primarily mucosal sites	Maturation, integrity, pathogen resistance	Many associations; few causal cattle studies
Bile transformation	Deconjugation and secondary modification	Bile-acid derivatives	Small and large intestine	Lipid handling and signalling	Emerging cattle-specific evidence

### From Taxonomic Composition to Active Function

Functional redundancy is scale dependent. Several organisms may carry genes annotated to the same broad pathway, yet differ in substrate range, kinetics, regulation and tolerance of low pH or inhibitory compounds. Redundancy at the level of ‘carbohydrate metabolism’ does not guarantee redundancy for degradation of crystalline cellulose or a particular hemicellulose linkage. High-level database categories can therefore exaggerate stability. Enzyme families, genomic context and measured products provide a more discriminating assessment [10, 52]. Metatranscriptomics offers a time-sensitive view of expressed genes. It can reveal that a low-abundance organism contributes disproportionately to transcription or that taxa with stable abundance alter their activity after feeding. RNA preservation is challenging, and ribosomal RNA depletion, sequencing depth and reference mapping affect results. Repeated time points are especially important in the rumen because transcription can respond rapidly to substrate pulses. A single pre-feeding sample should not be treated as the animal’s complete functional state [3, 53].

Metaproteomics detects enzymes that are more stable than transcripts and closer to catalytic function, but shared peptide sequences can prevent confident species assignment. Protein extraction from fibre-rich digesta is technically difficult, and abundant host or feed proteins can reduce microbial coverage. Metabolomics measures the integrated chemical outcome but cannot automatically assign microbial origin. Host cells, feed and multiple microbial pathways may produce the same metabolite. These limitations are reasons to combine methods, not reasons to select one as universally superior [11, 14]. Stable-isotope probing and flux approaches can test who uses a substrate and where carbon or nitrogen moves. Labelled cellulose, starch, amino acids, bicarbonate or urea can connect community activity to products and host absorption. Such methods are more demanding than sequencing but address the causal gap that reviewers increasingly identify in descriptive microbiome studies. Cultivation remains equally important because isolates and defined consortia allow direct testing of substrate use, cross-feeding and inhibitor sensitivity [10, 52].

Genome catalogues have changed what can be annotated. Stewart et al. [16] assembled 913 rumen microbial genomes, and Xie et al. [9] produced a broader ruminant GIT catalogue containing more than 10,000 metagenome-assembled genomes. Teseo et al. [27] expanded resources for the cattle large intestine. These catalogues improve read mapping and pathway reconstruction, but they remain biased towards organisms abundant enough to assemble and samples represented in the underlying datasets. Continued cultivation and sampling from underrepresented breeds, diets and regions remain necessary [3, 53]. Taxonomic profiles answer ‘who is detected’; they do not establish ‘what is occurring’. Two animals can differ in the relative abundance of individual taxa yet maintain similar VFA patterns because multiple genomes encode overlapping carbohydrate and fermentation pathways. Conversely, similar taxonomic profiles can exhibit different activity when substrate availability or redox state changes. Rumen studies connecting metagenomes, metatranscriptomes and metabolites show that pathway expression and metabolite patterns can track feed efficiency more directly than a single taxon [52]. A heritable microbial subset associated with host traits is also informative, but association and heritability do not alone prove that altering those organisms will reproduce the phenotype [54].

A rigorous multi-omics design begins with a defined causal question. Metagenomics identifies functional potential; metatranscriptomics tests whether genes are expressed; metaproteomics tests whether enzymes are present; metabolomics measures the resulting chemical environment; and isotope tracing or flux analysis tests reaction flow. These layers should be collected from matched samples and time points. Otherwise, apparent integration may compare different animals, compartments or physiological states. Network analysis can prioritise candidate interactions, but compositional correlations and shared responses to diet can create false edges [17]. Validation requires perturbation, culture or co-culture, targeted assays, and ultimately animal-level outcomes.

### Microbiome Modulation and Practical Outcomes

#### Feed Efficiency and Precision Nutrition

Feed efficiency is a composite phenotype affected by intake, digestion, maintenance, activity, tissue metabolism and product formation. Microbial fermentation influences several of these components, but residual feed intake and related measures can also correlate with body composition and behaviour. Studies linking microbiota to efficiency should use adequately sized cohorts, account for lactation stage and diet, and validate models in independent animals. Feature selection performed on the full dataset before cross-validation can substantially overestimate predictive performance [52, 54, 55]. Rumen and faecal biomarkers may provide complementary information. Rumen profiles are mechanistically closer to primary fermentation, while faecal samples are easier to collect repeatedly and may reflect substrate escape and distal responses. The relevant question is not which site is universally best, but whether its measurement improves a defined prediction beyond intake, diet, milk records, genetics and sensor data. Cost, turnaround time and sample stability are part of that evaluation [52, 54, 55].

Precision feeding currently relies mainly on feed analysis, intake estimates, milk composition, body condition, environmental data and production models. Microbiome measurements could eventually refine animal stratification or detect atypical responses, but sequencing is not yet a real-time control signal. Claims of commercial readiness should require prospective trials in which microbiome-informed decisions improve efficiency, health or emissions compared with existing practice. Wallace et al. [54] and Xue et al. [52] provide important biological foundations, not evidence that such systems are already routine [52, 54, 55]. Microbial features associated with feed efficiency could support stratification, biomarker discovery and diet design. Large genomic catalogues improve the annotation of previously unclassified functions [9, 16], and multi-omics studies can identify candidate pathways [52]. However, most applications remain at the research or model-development stage. Routine real-time metagenomics-guided ration formulation has not been demonstrated on commercial dairy farms. Current barriers include sampling delay, sequencing cost, batch effects, model transportability and the need to show added value over intake, milk, body-condition and diet measurements.

#### Methane Mitigation in Proportion

Mitigation outcomes should be expressed clearly as daily methane production, methane yield per unit of dry-matter intake, and methane intensity per unit of milk or weight gain. These measures answer different questions. Lower daily methane caused by reduced intake is not equivalent to improved fermentation efficiency, and lower intensity can occur through increased production without lower absolute emissions. Trials should report all relevant denominators together with intake and animal performance [51, 56, 57]. 3-NOP has one of the clearest target-based mechanisms among feed additives because it inhibits the terminal methyl-coenzyme M reductase step. The dairy-cow trial by Hristov et al. [56] showed persistent reductions without reduced milk production under the tested conditions. Magnitude varies with dose, diet and delivery, and responses should not be converted into a single universal percentage. Long-term adoption also depends on formulation, feeding system, approval and economics [58–60].

*Asparagopsis taxiformis* has produced very large methane reductions in specific beef-steer diets [60], but its bromoform content, stability, intake effects, residues, cultivation footprint and continuous delivery require evaluation. Extrapolation from a controlled beef study to grazing or dairy systems is not automatic. Lipids, tannins, nitrate, monensin and forage-quality strategies likewise differ in mechanism, consistency and risk. Synthesis reviews are more appropriate than isolated maximum responses when comparing options [51, 57, 51, 56, 57]. Microbial adaptation can weaken responses when communities bypass an inhibitor or restore hydrogen flow. Conversely, direct inhibition can persist if the target is difficult to circumvent and the compound is supplied consistently. Longitudinal community and activity measurements can distinguish adaptation from changes in intake or additive exposure. Environmental assessment should extend beyond enteric methane to feed production, manure, nitrogen losses and product output so that mitigation is not achieved by shifting burdens [58–60].

Methane mitigation is an important but not exclusive application. The inhibitor 3-nitrooxypropanol (3-NOP) directly targets methyl-coenzyme M reductase and can persistently reduce emissions in dairy cattle under tested conditions [56]. Meta-level evidence supports some dietary and management approaches but also shows substantial heterogeneity by dose, basal diet, production system and measurement method [51, 57]. Monensin effects are generally smaller and may diminish with time; red seaweed has produced large reductions in specific beef-steer trials, but intake, residue, formulation and long-term dairy applicability require context [60]. Reduction percentages should therefore be reported only with species, dose, duration and comparator, not as universal properties of an additive.

#### Gut Health, Barrier Function and Pathogen Resistance

Direct-fed microbials and fermentation products can act through multiple routes, including substrate competition, lactate utilisation, oxygen scavenging, cell-wall immune effects and changes in feed digestion. Results cannot be generalised from one strain or product to another. Jiang et al. [25] provides an example of pairing an intervention with ileal microbial and tissue measures, but stronger evidence requires replicated clinical or production endpoints and confirmation that the proposed microbial mediator changes before the host outcome [40, 41]. Pathogen resistance may involve exclusion from attachment sites, nutrient competition, bacteriocins, organic acids and immune priming. These functions are often strain specific, making genus-level abundance a poor surrogate. For food-safety applications, studies should measure pathogen load or shedding and consider whether an intervention changes virulence or antimicrobial-resistance genes. Reducing a pathogen’s relative abundance without changing its absolute shedding may have little practical value [25, 43, 44].

Intervention safety deserves the same attention as efficacy. Introducing live organisms requires identity, stability and antimicrobial-susceptibility assessment. Plant and marine bioactives can affect intake, liver function, residues or non-target microbes. Early-life interventions may influence immune development in ways that are not captured by short growth trials. A responsible microbiome strategy therefore includes dose-response work, withdrawal or persistence assessment and surveillance for unintended functional changes [40, 41]. Interventions can also target calf diarrhoea risk, epithelial maturation, colonisation resistance, nitrogen efficiency and recovery from dietary challenges. Probiotics, prebiotics and early nutritional strategies may alter these outcomes, but responses depend on strain, dose, age, diet and farm environment. A change in diversity is not inherently beneficial, and faecal taxonomic shifts should not substitute for clinical, barrier or production endpoints. Safety assessment must consider antimicrobial-resistance genes, opportunistic organisms and unintended shifts in metabolite production [25, 43, 44].

The evidence supporting major microbiome interventions and the cautions governing their interpretation are summarised in Table 3.

**Table 3:** Evidence-graded microbiome interventions and interpretive cautions

Intervention	Primary rationale	Evidence level	Supported interpretation	Key cautions	Sources
3-NOP	Direct enzyme inhibition of methanogenesis	Replicated in vivo cattle evidence	Often decreases enteric CH₄; magnitude depends on dose and diet	Persistence, delivery system, production response and regional regulation	[56, 51]
Diet composition/forage quality	Changes substrate, passage and H₂ partitioning	Established, context dependent	Can improve efficiency and alter CH₄ yield	Avoid confounding lower emissions with lower intake or fibre digestion	[51, 57]
Lipids	Suppress selected fermenters and provide alternative H sinks	Moderate-to-strong synthesis evidence	CH₄ reduction varies with fatty acid and dose	High inclusion can depress fibre digestion and milk fat	[51]
Monensin	Redirects fermentation and H₂ production	Meta-analytic evidence; adaptation possible	Usually modest and variable CH₄ effect	Not approved for all uses/regions; evaluate productivity and resistance stewardship	[51]
Condensed and hydrolysable tannins	Bind proteins and can suppress selected methanogens or hydrogen-producing microbes	Synthesis evidence; strongly source- and dose-dependent	Variable effects on CH₄, nitrogen use and digestion	High doses can depress intake and digestibility; chemical characterisation is essential	[59, 51]
*Asparagopsis taxiformis*	Bromoform-containing material inhibits methanogenesis	Strong effects in some beef trials; external validity incomplete	Large reductions reported in defined diets/doses	Composition, residues, intake, supply and long-term dairy evidence	[60]
Probiotics/prebiotics	Strain- and substrate-specific ecological modulation	Mixed and product-specific	Potential health or fermentation benefits	Do not generalise across strains; require clinical or production endpoints	[44]
Early-life diet/management	Shapes succession during a developmental window	Established short-term succession; long-term programming emerging	May influence rumen maturation and calf health	Adult persistence and causal production gains need longitudinal trials	[34, 35, 45]

### Research Priorities

A minimum reporting framework would substantially improve comparability. Studies should identify breed, age, sex, physiological stage, diet composition and processing, intake, health and antimicrobial history; describe the exact compartment and sample phase; report collection time relative to feeding, preservation and extraction; include negative controls; and deposit raw data and metadata. For intervention studies, dose, formulation, duration, adherence and washout should be explicit. These details determine whether results can be reproduced or synthesised [3, 11, 17].

Underrepresented production systems are a major gap. Current reference catalogues are dominated by a limited range of breeds, diets and intensively managed animals. Tropical forages, indigenous cattle, pasture systems and smallholder environments may expose microbes to different substrates and management pressures. Broader sampling is necessary both for biological understanding and to prevent prediction models from failing when transferred across regions [3, 11, 17].

Longitudinal anatomical sampling. Follow the same animals from birth through production, sampling multiple compartments where ethically feasible and recording diet, passage, host physiology and health.

Mucosa–digesta separation. Analyse paired luminal, particle-associated and mucosal communities so that epithelial interactions are not inferred from faeces.

Activity and flux. Combine DNA with RNA, proteins, metabolites, absolute microbial load and isotope or mass-balance approaches.

Hindgut methane quantification. Develop direct or validated partitioning methods rather than inferring emissions from methanogen abundance.

Causal validation. Test candidates using controlled perturbations, defined consortia where possible, and animal-level replication across farms.

Transparent evidence grading. Report species, age, diet, dose, duration, sampling site and measurement method; label hypotheses explicitly.

Translation criteria. Demonstrate that microbiome measurements improve prediction or decisions beyond inexpensive farm records before claiming precision-feeding readiness.

## Conclusion

The strongest tract-wide conclusion is not that all bovine GIT compartments are equally characterised, but that anatomical region imposes reproducible ecological structure on a sequential digestive system. The rumen remains the dominant and best-supported site of fermentation and methanogenesis. Evidence for downstream communities is sufficient to establish regional distinctness and plausible roles in residual fermentation, barrier biology and immune signalling, but often insufficient to quantify activity or production consequences. Early-life succession adds a developmental axis that is central to rumen maturation and mucosal immunity, while claims of permanent programming require longer follow-up. Practical progress depends on matching claims to evidence. Methane interventions should be judged by whole-animal emissions, productivity, persistence and safety; gut-health interventions by clinical and barrier outcomes; and precision-nutrition tools by incremental predictive value under commercial conditions. The immediate priorities are longitudinal multi-compartment studies, paired mucosa and digesta sampling, direct activity and flux measurements, and causal validation across farms. Rumen metagenomics and multi-omics have considerable potential to contribute to precision feeding by identifying microbial features associated with production efficiency, methane emissions and milk composition. At present, however, routine real-time metagenomics-guided ration formulation remains a research objective rather than a demonstrated commercial practice.

## Data Availability

No datasets were generated or analysed during the current study.
